# Village community mobilization is associated with reduced HIV incidence in young South African women participating in the HPTN 068 study cohort

**DOI:** 10.1002/jia2.25182

**Published:** 2018-10-17

**Authors:** Sheri A Lippman, Anna M Leddy, Torsten B Neilands, Jennifer Ahern, Catherine MacPhail, Ryan G Wagner, Dean Peacock, Rhian Twine, Dana E Goin, F Xavier Gómez‐Olivé, Amanda Selin, Stephen M Tollman, Kathleen Kahn, Audrey Pettifor

**Affiliations:** ^1^ Center for AIDS Prevention Studies University of California San Francisco CA USA; ^2^ MRC/Wits Rural Public Health and Health Transitions Research Unit (Agincourt) School of Public Health Faculty of Health Sciences University of the Witwatersrand Johannesburg South Africa; ^3^ Division of Epidemiology School of Public Health University of California Berkeley Berkeley CA USA; ^4^ School of Health and Society University of Wollongong Wollongong NSW Australia; ^5^ Wits Reproductive Health and HIV Research Institute University of the Witwatersrand Johannesburg South Africa; ^6^ Division of Epidemiology and Global Health Department of Public Health and Clinical Medicine Umeå Centre for Global Health Research Umeå University Umeå Sweden; ^7^ Sonke Gender Justice Cape Town South Africa; ^8^ School of Public Health University of Cape Town Cape Town South Africa; ^9^ Gillings School of Global Public Health University of North Carolina at Chapel Hill Chapel Hill NC USA; ^10^ INDEPTH Network Accra Ghana

**Keywords:** community mobilization, critical consciousness, HIV incidence, adolescents, social capital, South Africa

## Abstract

**Introduction:**

Adolescent girls and young women (AGYW) in South Africa bear a disproportionate burden of HIV. Community mobilization (CM), defined as community members taking collective action to achieve a common goal related to health, equity and rights, has been associated with increased HIV testing and condom use and has been called a ‘critical enabler’ for addressing the HIV epidemic. However, limited research has examined whether CM is associated with HIV incidence among AGYW.

**Methods:**

We examine the association of CM with incident HIV among AGYW (ages 13 to 21) enrolled in the HPTN 068 cohort in the Agincourt Health and socio‐Demographic Surveillance System, South Africa. This analysis includes 2292 participants residing in 26 villages where cross‐sectional, population‐based surveys were conducted to measure CM among 18‐ to 35‐year‐old residents in 2012 and 2014. HPTN 068 participants completed up to five annual visits that included an HIV test (2011 to 2016). Household‐level data were collected from AGYW parents/guardians and census data is updated annually. Mean village‐level CM scores were created using a validated community mobilization measure with seven components (social cohesion, social control, critical consciousness, shared concerns, organizations and networks, leadership and collective action). We used pooled generalized estimating equation regression with a Poisson distribution to estimate risk ratios (RR) for the association of village‐level CM score and CM components with incident HIV infection, accounting for village‐level clustering and adjusting for key covariates.

**Results:**

There were 194 incident infections over the follow‐up period. For every additional standard deviation of village‐level CM there was 12% lower HIV incidence (RR: 0.88, 95% CI: 0.79, 0.98) after adjusting for individual, household and community characteristics. CM components associated with lower HIV incidence included critical consciousness (RR: 0.88; CI: 0.79, 0.97) and leadership (RR: 0.87; CI: 0.79, 0.95); while not statistically significant, social cohesion (RR: 0.91; CI: 0.81, 1.01), shared concerns (RR: 0.90; CI: 0.81, 1.00), and organizations and networks (RR: 0.91; CI: 0.79, 1.03) may also play a protective role.

**Conclusions:**

These results suggest that having strong community social resources will reduce AGYW's risk of HIV acquisition. Work to mobilize communities, focusing on building social cohesion, shared concerns, critical consciousness, and effective and accountable leadership, can fortify prevention programming for AGYW.

## 1 Introduction

It is estimated that there are over 8500 new HIV infections per week among adolescent girls and young women (AGYW) ages 15 to 24 years in sub‐Saharan Africa 1. Within the region, South Africa has the largest epidemic; 5.6% of AGYW ages 15 to 19 are living with HIV, increasing to over 17.4% by ages 20 to 24 2. The steep rise in HIV incidence during this time is shaped by a critical period of human development marked by profound physical, cognitive, and social changes and developmental tasks (e.g. establishing identity, independence) that characterize the transition from adolescence to young adulthood 3, 4, 5, 6. Within this complex transition period, the sociocultural environment is likely to play a large role in shaping behaviours and risk 6, 7. In fact, adolescence has been labelled a period of “heightened sensitivity to sociocultural signals in the environment” 8 when the influence of peers and their school and community environments may play a greater role in determining HIV risk than at other stages of their lives 9, 10.

There is growing evidence that the social environment, inclusive of the physical surround and cultural context in which social relationships occur and people interact 11, shape health and health behaviours 12, 13, 14, 15, 16. For example, studies of community well‐being or a sense of community connectedness, social capital and social cohesion have demonstrated protective effects on early sexual debut and rates of sexually transmitted infections in the US 17, 18, 19, 20, 21. Studies in multiple contexts have also found that women who perceive their environments to be cohesive or who engage in community groups have better sexual health outcomes 22, 23, 24, 25, 26. There is also evidence that in the critical adolescent years, increasing social connection to and engagement within the community is associated with protective behaviour 27, 28. Prosocial involvement or participation in the community, including participation in school groups, athletics or sports clubs, religious groups, or arts and cultural groups, can provide young people with a sense of meaning, value, or belonging, and has been associated with lower levels of substance use, risky sexual behaviour and violence 29, 30, 31, 32, 33, 34, 35, 36, 37.

To shape and harness community well‐being to support young people in preventing HIV infection, it is critical to improve our understanding of the many facets of community ‘social health’ that may play a role in HIV. Currently, there is disagreement around which aspects merit focus and a lack of consensus on how to monitor and measure these components. In recent years, there has been a growing international focus on community mobilization (CM) for health, which UNAIDS has called a critical enabler for HIV programmes, or “an activity that is necessary to support the effectiveness and efficiency of basic programme activities” 38. To further efforts to engage and mobilize communities and understand which aspects of the social environment can facilitate improved health for young people, our team developed a conceptual framework and measure of community mobilization – a collection of community characteristics and processes that we hypothesize are collectively needed to improve health outcomes or behaviours 39, 40. These mobilizing components include: (1) a shared issue or concern that is the target of change; (2) community sensitization or building of critical consciousness; (3) an organizational structure with links to groups/networks; (4) leadership (individual and/or institutional); (5) collective activities/actions; and (6) community cohesion 39. We also measured a seventh component: social control, or the mutual expectation of community members to intervene for shared interests 41, 42. We previously developed and validated the Community Mobilization Measure (CMM) 40, and applied our measure in a population‐representative survey across 26 villages where longitudinal research with AGYW was underway. In this manuscript, we examine whether living in a community with higher levels of mobilization is associated with HIV incidence among AGYW and assess which community mobilization components are associated with reduced HIV incidence. As a result, this manuscript expands the focus of this special issue on community engagement theory and practice in research to a broader view of community mobilization for health, offering findings that can inform future directions for both complementary areas of study.

## 2 Methods

### 2.1 Setting and procedures

HPTN 068 took place in the high HIV‐prevalence district of Ehlanzeni, South Africa 2 within the rural Agincourt Health and socio‐Demographic Surveillance System (HDSS) site, where the Medical Research Council/Wits University Rural Public Health and Health Transitions Research Unit (Agincourt) conducts an annual census 43. HPTN 068 (NCT01233531) was a randomized trial of cash transfers conditional on school attendance among 2533 AGYW ages 13 to 20 residing in the Agincourt HDSS study area enrolled in grades 8 to 11 at local government (public) schools at the time of study enrolment (March 2011 to December 2012). Following informed consent procedures, cohort participants were randomized 1:1 to conditional cash transfer or to the control condition. In both arms participants completed an audio computer‐assisted self‐interview and HIV counselling and testing (HCT) at baseline and at up to three follow‐up visits during the 068 trial and an additional posttrial visit; follow‐up visits occurred approximately annually. Parents or guardians completed a computer‐assisted personal interview to gather household‐level data at baseline and each follow‐up visit during the 068 trial period. A detailed description of the 068 trial and cohort is published elsewhere 44, 45.

Simultaneous to the HPTN 068 trial, a community mobilization programme and research initiative was underway at the Agincourt HDSS site, with implementation of a CM intervention in 11 of 22 randomly selected villages in the area 46. The CM intervention, conducted in partnership with Sonke Gender Justice and carried out by a trained team of mobilizers and community volunteers, sought to address intersections around HIV risk and gender norms that contribute to gender‐based violence and power inequities, encouraging community members to examine how to make changes in both their own lives and in their communities through workshops and varied community activities. The intervention was evaluated using cross‐sectional surveys conducted prior to (n = 1181) and following (n = 1403) the two‐year intervention (2012 to 2014). Survey participants included randomly sampled adults, ages 18 to 35 years, with approximately 55 people in each community (or village) at both time points. The sampling frames for the surveys were the 2011 and the 2013 Agincourt HDSS annual census, respectively. Eligibility criteria for participation included: consent to participate in the survey, residence in the home, being 18 to 35 years of age, and having lived in the study village for the majority of the past 12 months. A detailed description of the survey sampling and procedures is previously published 46, as are trial results 47, 48. This manuscript utilizes the CM domain measures to understand aspects of the social environment that shape HIV risk among AGYW.

**Figure 1:**
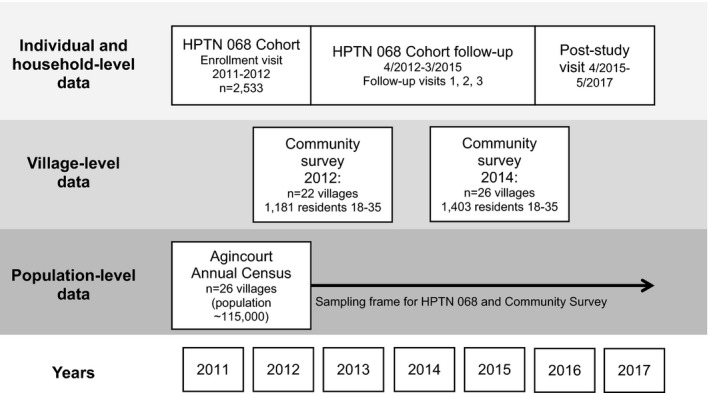
Study schematic of three contributing data sources and data collection timelines in Agincourt, South Africa.

Institutional Review Board approval for HPTN 068, for the community surveys, and for merging the data sources for these analyses was obtained from the University of North Carolina at Chapel Hill (UNC) and the University of the Witwatersrand Human Research Ethics Committee. The University of California‐San Francisco also approved the community surveys and protocols for merging data. The data sources merged for this analysis is displayed in Figure 1.

### 2.2 Measures

**Figure 2:**
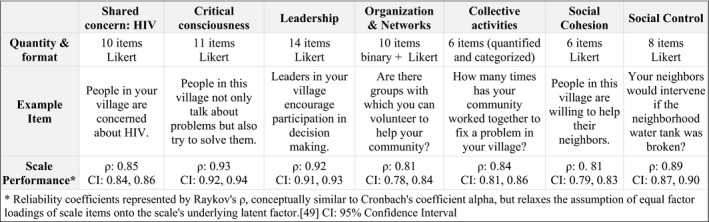
Community mobilization domains and measures.

We collected quantitative measures of CM domains in both the 2012 and 2014 community surveys. The community mobilization measure (CMM) is composed of seven domains (Figure 2). Questions regarding a shared concern about HIV/AIDS are designed to capture whether members of the community (1) define HIV as an important, problematic and mutable issue; (2) discuss and are aware of the impacts of HIV in their village; and (3) believe they can work together to improve outcomes. The shared concern scale is the only topic‐specific scale, the other subscales refer to general community qualities, not specific to HIV. The scale for critical consciousness is designed to capture whether members of the community are undergoing processes of critical reflection and dialogue about their circumstances and ways to address injustices. Questions about leadership capture leadership capacity, diversity, responsiveness, accessibility and support of collective decision‐making. Questions regarding organizations and networks are designed to capture the existence and influence of community‐based organizations, groups and networks that can serve as a resource in mobilizing – both for exchange and diffusion of ideas and as a structure that can be utilized for community organizing. Questions regarding collective action are designed to capture the presence, breadth and quantity of collective activities in the villages aimed at social change. Questions about social cohesion and social control capture the level of working trust and mutual expectation to intervene for the common good, as originally theorized by Sampson and colleagues 41, 42. Based on formative work, responses included 3‐point Likert scales, with responses including “agree a lot, somewhat agree, do not agree at all” for all domains except social control, which included responses of “very likely, somewhat likely, unlikely,” and organizations and networks which assessed whether organizations existed and if they were “very important, a little important, or not important” in the community. We aggregated individual responses on the surveys into mean community mobilization scores and domain scores for each village, with higher scores indicating increasing amounts of each domain (e.g. more mobilization). The measures, their performance (reliability coefficients 49) on the 2012 survey and example items are described in Figure 2 and reported on extensively in a previous publication 40.

HIV status in the AGYW 068 cohort was determined by conducting parallel HIV rapid tests in the field using the Determine HIV‐1/2 test (Alere Medical Co, Matsudo‐shi, Chiba, Japan) and Uni‐gold Recombigen HIV test (Trinity Biotech, Bray, County Wicklow, Ireland). If both HIV rapid tests were non‐reactive, no further testing was done at that study visit. If one or both tests were reactive or positive, confirmatory HIV testing was conducted using a western blot assay. Quality control of HIV diagnosis was performed at the HPTN Laboratory Center to confirm baseline HIV status and incident HIV infections.

Covariates of interest at the individual level included age at study entry and 068 study arm as well as a number of time varying covariates including study visit, current educational status (in school or graduated vs. not attending or dropped out), and family household assets (operationalized as the total number of durable goods from a list of 27 items each household owned). At the community level, covariates came from the census data. We explored mean years of education in the community, the proportion of the community composed of permanent residents, and the mean socio‐economic status (SES) derived from a list of household assets, access to water, housing material and owned livestock, with higher scores indicating more assets. We noted instability in regression coefficient results when using the original community characteristics variables due to multicollinearity. We therefore used the ‐pca‐ command in STATA with a varimax rotation (orthogonal transformation) to repartition the total variance of the three correlated variables into linearly uncorrelated principal components, which were included as control variables in the analyses described below. While component scores are less interpretable than original variables, this approach allows us to remove potential confounding of community SES, education, and residency. Communities with higher scores on the combined measure were more highly educated, wealthier and had fewer permanent residents (were more mobile). We also included village intervention randomization assignment from the community mobilization study (2012 to 2014) as a covariate *a priori*, though prior analyses have indicated that scores did not differ between the intervention and control communities (i.e. the intervention did not impact CM scores) 47.

### 2.3 Analysis

To assess how a community's level of mobilization is related to HIV incidence among AGYW, accounting for individual, household and community‐level factors, we merged data from the 068 participant and household surveys, the two community surveys, and the census data. The merging process was conducted such that each HPTN visit was assigned the most recent village data, including CM scores. As a result, the data structure ensures community (exposure) data precedes HIV outcome data and preserves temporality. For this analysis, we have restricted our study population to young women who were HIV‐negative at entry (78 with prevalent HIV infection at baseline were excluded), and to those who lived in villages included in the community surveys (n = 159 excluded due to no community data). Finally, we excluded four participants who became HIV infected prior to having community survey data, in order to ensure temporal ordering (n = 4).

We used pooled generalized estimating equation (GEE) regression with a Poisson distribution to estimate the risk ratios (RR) of incident HIV infection among AGYW, adjusting for relevant confounders and addressing village‐level clustering via robust Huber–White cluster‐adjusted standard errors. Community mobilization scores were standardized using the pooled standard deviation for the 2012 and 2014 surveys (to ensure comparability) and included in multivariate analyses such that the risk ratio represents the difference in HIV incidence associated with a one standard deviation increase in each community measure/score. Bivariate Poisson regression was used to determine the association between the independent variables and HIV incidence. Covariates were included in the adjusted analysis if they were significant at the 0.1 level in the bivariate analysis or were selected *a priori* based on the literature.

## 3 Results

In total, 2292 AGYW living across 26 communities were included in this analysis. At enrolment, participants had a mean age of 15.5 years and 100% were in school, with just over 26% reporting being sexually active and 3% reporting engaging in transactional sex. (Table 1) By the end of the follow‐up period, 88% were either graduated from high school (matric) or still in school (12% had dropped out/never graduated) and 58% reported ever having sex with 24% reporting engaging in transactional sex in the past 12 months. There were 194 incident infections over the follow‐up period. Community demographics did not change substantively over time. Community mobilization domain scores varied slightly over time with differences that were not statistically significant. (Table 1).

**Table 1:** Baseline characteristics of HIV‐negative adolescents girls and young women enrolled in HPTN 068 (n = 2292) and their communities (n = 26)

Participant characteristics	Baseline (n = 2292) n (%)	By end of follow‐up (n = 2225) n (%)
Mean age at entry into 068 (SD)	15.5 (1.6)	–
In school or graduated	2229 (100)	1961 (88.1)
Any sexual intercourse	613 (26.8)	1299 (58.4)
Have a sexual partner ≥5 years older in past 12 months^a^	119 (5.2)	330 (14.8)
Engage in transactional sex in past 12 months^a^	72 (3.1)	548 (24.6)
Condomless sex in last three months^a^	189 (8.3)	699 (31.4)
HIV status		
HIV negative	2292 (100)	2031 (91.3)
HIV positive	0	194 (8.7)
Drink alcohol once a month or more	117 (5.10)	432 (19.4)
Mean number of household assets, (SD) (asked about 27 durable goods)	14.03 (0.06)	15.56 (0.07)
**Community characteristics** b	**Unweighted mean (SD)** 2012	**Unweighted mean (SD)** 2014
Mean years of education	6.08 (0.61)	6.79 (0.49)
% permanent residents	62.36 (4.23)	59.81 (3.81)
Mean SES asset score	0.09 (0.54)	0.09 (0.52)
**Community mobilization** c	**Weighted mean (SD)** 2012	**Weighted mean (SD)** 2014
Total community mobilization score	2.22 (0.12)	2.15 (0.11)
Social cohesion	2.34 (0.15)	2.48 (0.16)
Social control	1.96 (0.17)	1.31 (0.13)
Critical consciousness	2.46 (0.15)	2.49 (0.17)
Shared concerns (around HIV)	2.13 (0.10)	2.23 (0.18)
Leadership	2.10 (0.19)	2.19 (0.22)
Organizations and networks	0.98 (0.19)	0.84 (0.17)
Collective action	1.60 (0.28)	1.16 (0.08)

a Among sexually active participants.

b Data from Agincourt Health and socio‐Demographic Surveillance System census.

c Data from community surveys.

Association of the overall village community mobilization score with incident HIV infection, adjusting for individual and community‐level covariates is presented in Table 2. Community mobilization village score was protective against HIV incidence in both unadjusted (RR: 0.77; CI: 0.65, 0.91) and adjusted (RR: 0.88; CI: 0.79, 0.98) analyses, such that for every additional standard deviation in village‐level CM there was a 12% lower HIV incidence after adjusting for age, study visit, education, household assets, 068 and CM randomization arms and community characteristics.

**Table 2:** Unadjusted and adjusted risk ratios (RR) of HIV among adolescent girls and young women enrolled in HPTN 068 (N = 2292)

Characteristics	Unadjusted RR (95% CI)	Adjusted^a^ aRR (95% CI)
**Individual level**		
Age at baseline	1.24 (1.15, 1.33)***	1.19 (1.13, 1.26)***
Study visit (first follow‐up visit is reference)		
Second follow‐up	1.27 (0.74, 2.19)	1.32 (0.76, 2.27)
Third follow‐up	1.67 (0.95, 2.95)	1.59 (0.95, 2.65)
Post‐intervention visit	4.85 (3.24, 7.25)***	3.38 (2.18, 5.24)***
Currently enrolled in school or graduated high school	0.17 (0.11, 0.28)***	0.50 (0.33, 0.77)**
Mean number of household assets	1.02 (0.99, 1.04)	1.00 (0.98, 1.03)
HPTN 068 intervention arm – cash transfer versus control	1.04 (0.77, 1.41)	1.09 (0.76, 1.55)
**Community level**		
Community mobilization	0.77 (0.65, 0.91)**	0.88 (0.79, 0.98)*
Community characteristics^b^	1.24 (1.15, 1.34)***	1.10 (1.02, 1.19)*
Community mobilization arm intervention village versus control	0.98 (0.74, 1.30)	0.91 (0.73, 1.14)

*P < 0.05, **P < 0.01, ***P < 0.001.

^a^Model adjusted for all other covariates in the table.

^b^Community characteristics is a collated measure of three community‐level variables (mean years of education, mean socio‐economic status asset score, and proportion of the community who are permanent residents).

We also explored the association between individual CM domains and incident HIV infection, in order to determine whether particular domains might be driving the association with HIV incidence among AGYW in the cohort (Table 3). While all CM domains with the exception of social control demonstrated a protective association, only critical consciousness (RR: 0.88; CI: 0.79, 0.97) and leadership (RR: 0.87; CI: 0.79, 0.95) reached statistical significance. Social cohesion (RR: 0.91; CI: 0.81, 1.01), shared concerns (RR: 0.90; CI: 0.81, 1.00), and the organizations and networks domain (RR: 0.91; CI: 0.79, 1.03) demonstrated similar magnitude of protective effects but did not reach statistical significance.

**Table 3:** Adjusted risk ratios (RR) of HIV incidence among adolescent girls and young women as a function of village mean community mobilization domain scores

CM domain	Adjusted^a^aRR (95% CI)
Social cohesion	0.91 (0.81, 1.01)
Social control	1.05 (0.96, 1.15)
Critical consciousness	0.88 (0.79, 0.97)*
Shared concerns (around HIV)	0.90 (0.81, 1.00)
Leadership	0.87 (0.79, 0.95)**
Organizations and networks	0.91 (0.79, 1.03)
Collective action	0.96 (0.82, 1.13)

*P < 0.05, **P < 0.01, ***P < 0.001.

^a^Adjusting for age at baseline, time, education, household assets, 068 intervention arm, community mobilization intervention arm, and community characteristics.

## 4 Discussion

We set out to understand the role of community mobilization in incident HIV among adolescent girls and young women living in rural communities in northeastern South Africa. We found that community mobilization, which is in essence a collection of different facets of community social resources, is associated with lower HIV incidence among AGYW longitudinally. To our knowledge, this is one of the first quantitative explorations of whether community‐level (in this case village‐level) social characteristics are protective against HIV in AGYW. We also noted which components of community mobilization contribute to the protective association, finding strong evidence for critical consciousness and leadership and suggestive evidence that social cohesion, shared concerns around HIV, and organizations and networks may also be protective against HIV infection. Overall findings indicate that AGYW experience reduced HIV infection in villages where residents feel connected, dialogue and address their circumstances, consider HIV an important community issue and have leadership that is present and accountable.

While community mobilization has been neither previously associated with reduced HIV incidence among adolescents nor empirically measured at a community level in a sub‐Saharan African context, studies in the United States have demonstrated health benefits of social capital, which is a related construct. Social capital characterizes the social resources and organization inherent in a group, including trust, norms and networks that facilitates coordination and benefits group members 29, 50. Social capital is most often operationalized as participation or civic engagement in community organizations and at times measured by community bondedness – making it similar to the cohesion and organizations and networks measures, included in our CMM. Research on social capital has demonstrated protective associations with sexually transmitted infections among youth in ecological (state and community‐level) studies 18, 51. Further, research conducted in the United States has noted protective associations of perceived social cohesion (most often measured as trust and closeness in a community) or social control (most often measured as expectations of reciprocity) with prevalence of sexually transmitted infections among youth 17, 19, 51 and early sexual debut 20, 21, 52. There has been less exploration of these constructs in sub‐Saharan Africa, with little previous research on community social resources measured at the community level and its impact on adolescent health. Studies of social capital and social cohesion at the individual‐level among adult women in Africa have yielded mixed results – some protective for HIV 26, 53 and some not 25.

To our knowledge, this is the first study to consider which aspects of community social resources are protective against HIV infections in young people, which is needed to guide future intervention work. We found strong evidence that critical consciousness and leadership are protective and additional suggestive evidence (not reaching statistical significance) that social cohesion, shared concerns around HIV, and organizations and networks may also be protective. These findings are consistent with the literature on establishing critical consciousness as a means to improve health and well‐being 54, 55, 56, and the extensive literature (cited above) on how cohesive communities and social trust can play a large role in fortifying community health. Furthermore, the HIV Competent Community initiatives 57 have provided insights into the role of critical consciousness as a means for communities to identify and resolve problems, with building of shared understandings and identities and collaborative partnerships playing a complementary role 54, 58, 59. Notably, we did not find an association with collective action, though between‐village variability in the later survey was likely insufficient to detect an association.

We also found a strong protective association between higher village leadership scores and reduced HIV incidence. Investigations of what is needed for successful health promotion have proposed that skilled, accountable, flexible and inclusive leadership and leadership networks (including coalition building) are essential in fostering structural change and strong health programming in the United States 60, 61, 62 and abroad 63, with poor or authoritarian leaders negatively impacting HIV community capacity 64. This study is, however, among the first to associate a quantitative measure of community‐rated quality of leadership (including items on community leaders’ capacity, diversity, responsiveness, accountability, accessibility, and support of collective decision‐making) with reduced HIV infections.

Finally, our results imply that having more local availability and higher levels of community engagement with organizations and networks (e.g. women's groups, cultural groups, school or youth groups, sports organizations and other groups seeking to support the community), might play a protective role. There are multiple pathways that a community with more engaged residents could be protective for youth, including building more opportunities for youth to get involved and participate themselves in community groups as well as instilling norms of engagement. Indeed, adolescent involvement in sports, clubs, and other organizations (either in school or extracurricular groups) can provide young people with a sense of meaning or belonging 37 and has been associated with better health outcomes, including improved sexual health 30, 33, 35, 36. Youth engagement in activities would also bring increased social contact with other youth, and therefore could also imply more protective sexual networks with less time to venture outside of those safer networks (for example with older partners who are more likely to be infected) 65. It is also possible that this domain is synergistic with leadership, in that communities with more accountable and inclusive leadership may also have more opportunities for organizations and networks to thrive, both of which contribute to (and are fortified by) having a more engaged citizenry.

While this study is among the first explorations of how community social factors may influence HIV infection among AGYW, we cannot comment on how these same community characteristics shape HIV incidence among adolescent boys and young men, who may benefit differently from community social resources. We have assessed each of the community mobilization domains separately and in a combined measure, but cannot yet comment on the interplay of these components in terms of temporal relationships between domains or complementarity and synergy in impacting AGYW outcomes, though this will be the topic of future study. Finally, though the CM measures in this study have undergone extensive validation, any measure of complex latent constructs will be imperfect.

Increased understanding of what it is about living in a more mobilized community that is protective will help lay the foundation for programming to address and enhance protective community social characteristics and provide an environment that enables risk reduction and optimizes HIV prevention for the broadest population 66, 67. Unfortunately, a great deal of HIV‐related programming remains focused on individual behaviour change and does not aim for structural change or engage in community mobilization, which may often lie beyond the comfort zone of health and research funding mechanisms and beyond their programme time horizons. There is a burgeoning movement for community engagement in large research initiatives, often conducted in service to biomedical trials to inform and involve communities through public education, outreach, and community advisory boards, but can also include broader participatory goals 68, 69. These efforts, based on Good Participatory Practice 70, also need to be distinguished from what we refer to as community mobilization. While the evolving field of community engagement can lead to improved community involvement in research and potentially to improved utilization of outcomes, which is laudable and important, this focus is unlikely to bring about sustained improvements in HIV outcomes without a purposeful emphasis on broad community capacity building. Community mobilization, in our view, is not about recruitment or motivating people to participate in research, but has at its core the building of community social resources to address inequities, disparities, and injustices and for communities to build their own responses to health, in this case HIV. Its purpose is not to facilitate research or to empower a few, but to build a collective community response 71. Nonetheless, understanding the community social resources, and to what extent communities may be mobilized, can serve as an important tool to inform good participatory practice, both as an indicator for heightened vigilance or more considerable resource provision in less mobilized communities and as a marker to expect intensified community involvement in highly mobilized communities. Though extensive CM programming may be beyond the purview of most biomedical trials, ways to factor in community building and fortification of social resources should be sought whenever such programming is feasible in order to ensure the broadest impact possible.

## 5 Conclusions

In the context of the persistent HIV epidemic among AGYW, insufficient attention has been paid to the community context and how communities might be strengthened to support prevention for young people. Our findings are among the first in sub‐Saharan Africa to draw a direct link from community social context in the form of mobilization domains to AGYW's HIV acquisition. Work to mobilize communities, focusing on fostering social cohesion, promoting shared concerns around critical health issues, generating dialogue and capacity building for critical consciousness, and encouraging engaged and accountable leadership, including availability and partnerships with networks and organizations, can fortify prevention programming for AGYW. Seeking to strengthen these community traits should not be an afterthought, but a conscious piece of HIV prevention and care programming for young people.

## Competing interests

We declare no conflicts of interest.

## Authors’ contributions

SAL, AP, TBN, JA and KK conceived this study. AP, KK, CM, SMT, FXGO, RT, RGW and AS were responsible for HPTN 068 design and implementation; SAL, AP, CM, DP, SMT, RGW, RT, AS, FXGO and KK were responsible for community mobilization study design and implementation; AML and DEG were responsible for data merging and management. AML led the analysis with assistance from TBN, SAL, JA and DEG. SAL and AML wrote the paper. All authors read and approved the final manuscript.
